# Development of a nomogram model for predicting acute stroke events based on dual-energy CTA analysis of carotid intraplaque and perivascular adipose tissue

**DOI:** 10.3389/fneur.2025.1566395

**Published:** 2025-03-11

**Authors:** He Zhang, Juan Long, Chenzi Wang, Xiaohan Liu, He Lu, Wenbei Xu, Xiaonan Sun, Peipei Dou, Dexing Zhou, Lili Zhu, Kai Xu, Yankai Meng

**Affiliations:** ^1^Department of Radiology, The Affiliated Hospital of Xuzhou Medical University, Xuzhou, China; ^2^College of Medical Imaging, Xuzhou Medical University, Xuzhou, China; ^3^Jiangsu Medical Imaging and Digital Medical Engineering Research Center, Xuzhou Medical University, Xuzhou, China; ^4^Department of Radiology, Jiawang District People's Hospital, Xuzhou, China; ^5^Department of Medical Imaging, Xuzhou New Health Hospital, Xuzhou, China

**Keywords:** dual-energy CT, CT angiography, carotid plaque, perivascular fat, acute stroke

## Abstract

**Objective:**

To evaluate the predictive value of dual-energy CT angiography (DECTA) parameters of carotid intraplaque and perivascular adipose tissue (PVAT) in acute stroke events.

**Methods:**

A retrospective analysis was conducted using clinical, laboratory, and imaging data from patients who underwent dual-energy carotid CTA and cranial MRI. Acute cerebral infarctions occurring in the ipsilateral anterior circulation were classified as the symptomatic group (STA group), while other cases were categorized as the asymptomatic group (ATA group). LASSO regression was employed to identify key predictors. These predictors were used to develop three models: the intraplaque model (IP_Model), the perivascular adipose tissue model (PA_Model), and the nomogram model (Nomo_Model). The predictive accuracy of the models was evaluated using receiver operating characteristic (ROC) analysis, calibration curves, and decision curve analysis. Statistical significance was defined as *p* < 0.05.

**Results:**

Seventy-five patients (mean age: 68.7 ± 8.7 years) were analyzed. LASSO regression identified seven significant variables (IP_Zeff, IP_40KH, IP_K, PA_FF, PA_VNC, PA_Rho, PA_K) for model construction. The Nomo_Model demonstrated superior predictive performance compared to the IP_Model and PA_Model, achieving an area under the curve (AUC) of 0.962, with a sensitivity of 95.8%, specificity of 82.4%, precision of 82.6%, an F1 score of 0.809, and an accuracy of 88.0%. The clinical decision curve analysis further validated the Nomo_Model’s significant clinical utility.

**Conclusion:**

DECTA imaging parameters revealed significant differences in carotid intraplaque and PVAT characteristics between the STA and ATA groups. Integrating these parameters into the nomogram (Nomo_Model) resulted in a highly accurate and clinically relevant tool for predicting acute stroke risk.

## Introduction

Stroke remains the leading cause of cardiovascular disease-related mortality in China (1). Among the numerous risk factors, vulnerable carotid artery plaques on the ipsilateral side are key contributors to acute stroke incidents (2). Studies have shown that active inflammation, the presence of a large lipid-rich necrotic core (LRNC), and intraplaque hemorrhage (IPH) are central in promoting plaque instability (3). Moreover, perivascular adipose tissue (PVAT) plays a significant role in enhancing plaque vulnerability by secreting pro-inflammatory cytokines that accelerate atherosclerosis (4, 5).

The vulnerability of carotid plaques has been extensively evaluated using high-resolution MRI, which provides significant value (6–8). However, MRI is constrained by long scan times, high costs, and patient compliance issues. Consequently, researchers have increasingly turned to CT angiography (CTA) to investigate the relationship between carotid plaques and acute stroke, yielding promising results (9, 10). Traditional CTA struggles to differentiate plaque types, limiting its ability to assess plaque vulnerability accurately. In contrast, dual-energy CT (DECT), which utilizes X-rays at varying energies, offers an innovative approach. Compared to MRI, DECT provides a faster, more affordable alternative with comparable or superior diagnostic capabilities, particularly for assessing carotid plaques and perivascular adipose tissue. Additionally, DECT enhances tissue characterization, enabling more precise identification of plaque components and improving stroke risk prediction.

Through advanced post-processing, DECT generates virtual non-contrast (VNC) images, fat fraction (FF), iodine concentration (IC), electron density (Rho), effective atomic number (Zeff), and the slope of the energy spectrum curve (K). Each of these parameters provides insights into material composition changes to varying extents (11–13). While some research has applied DECT to detect symptomatic carotid plaques (14, 15), its potential to predict acute stroke events by assessing fat content within and surrounding carotid plaques remains to be fully understood.

Thus, this study aims to evaluate the predictive value of dual-energy CTA (DECTA) parameters of carotid intraplaque and PVAT for the occurrence of acute stroke events.

## Methods

### Patients

This retrospective study has been approved by the Medical Ethics Committee of the Affiliated Hospital of Xuzhou Medical University, and the Institutional Review Board of this institution has waived the requirement for informed consent. All methods are in compliance with relevant ethical and regulatory requirements and adhere to the principles outlined in the Declaration of Helsinki.

This study enrolled 75 consecutive patients between January 2023 and August 2024, all of whom presented with neurological symptoms and/or exhibited positive findings during physical examinations. Each participant underwent dual-energy carotid CTA and cranial MRI.

Patients were eligible for inclusion based on the following criteria: (1) the interval between carotid CTA and cranial MRI did not exceed three days; (2) the presence of measurable carotid plaques (either unilateral or bilateral); (3) identifiable PVAT (either unilateral or bilateral); and (4) the absence of measurable atherosclerotic plaques in the ipsilateral anterior or middle cerebral arteries.

Patients were excluded for the following reasons: (1) poor image quality of CTA or MRI due to significant artifacts that would preclude post-processing analysis; (2) evidence of intracranial large vessel disease in the anterior circulation (e.g., stenosis or dissection); (3) underlying autoimmune vasculitis or intracranial vascular malformations; or (4) changes following stenting of the common carotid, internal carotid, or intracranial large arteries.

### DECTA scanning protocol

Dual-energy head and neck CTA scans were conducted using a Siemens third-generation dual-source CT scanner (SOMATOM Driver), covering the range from the aortic arch to the vertex of the skull. The scanning parameters included tube voltages of 80 kV and 140 kV; tube currents of 200 mAs and 100 mAs; a pitch of 1.1; a reconstruction layer thickness of 0.6 mm; an interval of 0.6 mm; a convolution kernel of Br40; and a rotation time of 0.5 s.

The contrast agent utilized was a non-ionic iodine medium, Iohexol (produced by Nanjing Zhengda Tianqing Pharmaceutical; trade name: Qinglidai; concentration: 100 mg/mL). A high-pressure injector (Medrad Stellant, Bayer AG) administered the contrast agent at a dosage of 1.0–1.2 mL/kg body weight, at an injection rate of 4.0 mL/s, followed by a 30 mL saline flush.

### Cranial MRI scanning protocol

Cranial MRI scans were performed using a GE 3.0 T magnetic resonance scanner (SIGNA Architect) and a Toshiba 1.5 T scanner (Vantage Elan). For the GE 3.0 T diffusion-weighted imaging (DWI) sequence, the scanning parameters were as follows: repetition time (TR) of 3,966 ms; echo time (TE) of 75.9 ms; slice thickness of 5 mm; spacing of 1.5 mm; field of view (FOV) of 240 mm × 240 mm; and a b-value of 1,000 s/mm^2^. For the Toshiba 1.5 T DWI sequence, parameters included: TR of 3,291 ms; TE of 100 ms; slice thickness of 6 mm; spacing of 1 mm; FOV of 240 mm × 240 mm; and a b-value of 800 s/mm^2^.

### Dual-energy carotid CTA image analysis

Two senior radiologists, each with over 15 years of experience in CTA post-processing, conducted the analysis of head and neck CTA images using a Siemens workstation (Syngo. Via VB40B). Regions of interest (ROIs) were strategically placed in the most prominent slices of the carotid plaques and PVAT gaps under the Liver VNC, Rho/Z, and Mono E modes.

One ROI was positioned within the plaque to encompass the entire structure in a single slice, ensuring the exclusion of high-density contrast medium effects. Two additional ROIs were placed in the PVAT gaps, ensuring each was located more than 1 mm from the edge of the carotid artery and surrounding tissues, in accordance with the measurement methodology proposed by Baradaran et al. (9), while avoiding surrounding soft tissues or small penetrating vessels. The average values from the two ROIs were subsequently utilized for statistical analysis. In cases of disagreement, the radiologists consulted to achieve consensus.

To resolve any disagreements between the two senior radiologists, a consensus-based approach was adopted. Both radiologists independently reviewed the images and identified the ROIs of the carotid plaques and PVAT gaps. If discrepancies in their measurements or interpretations arose, they engaged in direct consultation to reach a final decision. This collaborative process, leveraging both radiologists’ extensive experience, ensured that any conflicting views were thoroughly discussed and resolved. This approach ensured that both radiologists were aligned on the criteria for image analysis, improving the reliability and reproducibility of the measurements.

The DECTA parameters for the carotid plaque (Intraplaque, IP_) and perivascular adipose tissue (PA_) encompassed fat fraction (FF), virtual non-contrast (VNC) values, iodine concentration (IC), electron density (Rho), effective atomic number (Zeff), energy spectrum curve, and corresponding CT values for 40 keV (40KH) and 90 keV (90KH), as well as the slope of the intraplaque energy spectrum curve K. The formula employed is K _HU/keV_ = (90KH–40KH) / 50 _keV_ (Supplementary Figure S1). FF refers to the proportion of fat tissue within the plaque or surrounding perivascular adipose tissue, offering insights into the composition and stability of the plaque.

### Assessment of the acute cerebrovascular events

MRI images of patients’ brains were evaluated by two senior radiologists specialized in neuroimaging, using a Picture Archiving and Communication System (PACS). According to established guidelines, lesions exhibiting significantly high signals on diffusion-weighted imaging (DWI) and significantly low signals on the apparent diffusion coefficient (ADC) sequence are defined as acute cerebrovascular events (16).

**Figure 1:**
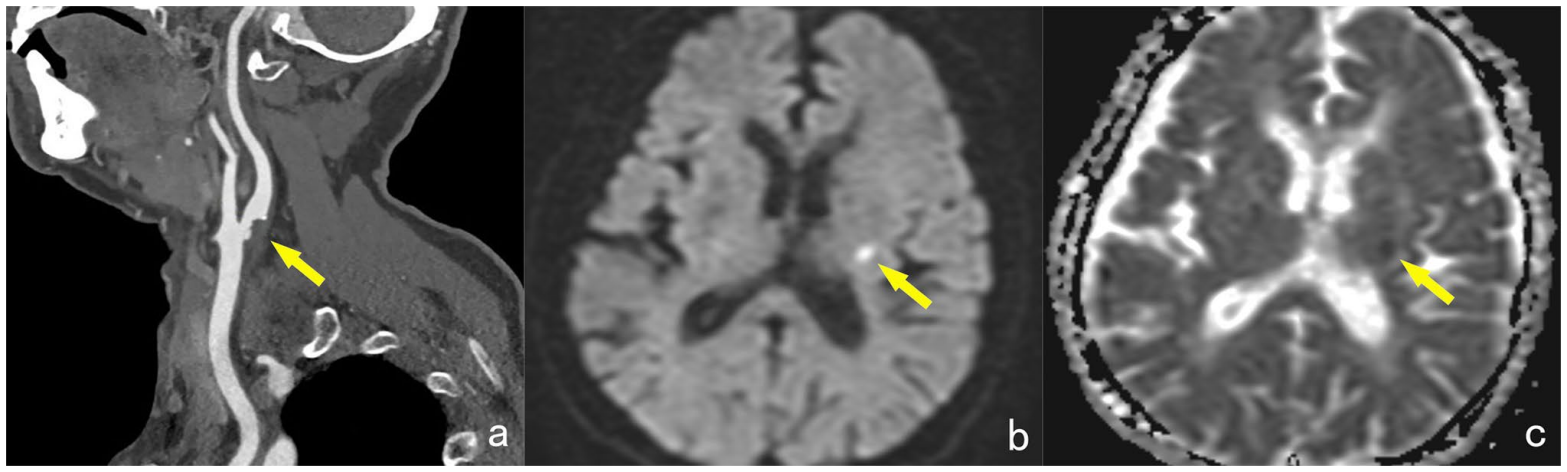
A 65-year-old female, showing a non-calcified plaque (yellow arrow) at the starting point of the left internal carotid artery on DECTA, with a narrowing degree of approximately 45% **(a)**. MRI examination reveals patchy high signals (yellow arrow) in the contralateral basal ganglia region on the DWI sequence **(b)**. The ADC map shows low signals (yellow arrow) in the lesion, with an ADC value of approximately 0.549 × 10^–3^ mm^2^/s, diagnosed as acute cerebral infarction and classified into the STA group **(c)**.

Acute cerebral infarctions occurring in the anterior circulation supply area on the same side are classified as the symptomatic group (STA group), while those without acute infarction in the same-side anterior circulation supply area are classified as the asymptomatic group (ATA group) (Figure 1).

### Clinical data collection

Clinical data were meticulously retrieved from the case management system by a radiologist (C.S) specializing in cardiovascular imaging. We retrospectively gathered comprehensive data, including gender, age, body mass index (BMI), history of hypertension, diabetes, transient ischemic attack (TIA), smoking history, alcohol consumption, and serum biomarker levels (total cholesterol, triglycerides, high-density lipoprotein, low-density lipoprotein). The interval between the collection of clinical laboratory data and CTA examinations did not exceed one week.

### Statistic

Data processing was conducted using SPSS version 23.0 and R software packages, including pROC, rms, glmnet, regplot and ggplot2. The normality of continuous variables was assessed using the Kolmogorov–Smirnov test. Continuous variables that followed a normal distribution were presented as (x ± s), while those with a non-normal distribution were expressed as M (Q1, Q3). Categorical variables were represented as percentages (%).

Comparisons of qualitative data between groups were performed using the χ^2^ test, whereas the *t*-test was applied for continuous data. For non-normally distributed data, the Mann–Whitney *U* test was used. Univariate variables with *p* < 0.05 were included in LASSO analysis, and selected variables were incorporated to construct the intraplaque model (IP_Model), the perivascular adipose tissue model (PA_Model) and the nomogram model (Nomo-Model). The models performance were evaluated using ROC analysis, calibration curve, clinical decision curve analysis and compared with the DeLong test. A *p* value of <0.05 was considered statistically significant.

### Data availability

The datasets used and/or analysed during the current study are available from the corresponding author on reasonable request.

## Results

### Comparison of clinical characteristics between the STA and ATA groups

No statistically significant differences were observed in clinical characteristics between the STA and ATA groups (all *p* values >0.05) (Table 1).

**Table 1:** Clinical data for STA and ATA groups (*N* = 75).

Clinical data	STA (*n* = 24) (%)	ATA (*n* = 51) (%)	*P* value
Male	16 (66.70)	36 (70.60)	0.731
Age (years)	68 (61.00, 74.50)	71 (64.50, 76.00)	0.481
BMI (kg/m^2^)	23.82 ± 2.89	25.18 ± 3.41	0.960
History of hypertension			0.370
Yes	16 (66.70)	39 (76.50)	
No	8 (33.30)	12 (23.50)	
History of diabetes			0.057
Yes	3 (12.50)	17 (33.30)	
No	21 (87.50)	34 (66.70)	
History of coronary heart disease			0.884
Yes	3 (12.50)	7 (13.70)	
No	21 (87.50)	44 (86.30)	
History of TIA			0.316
Yes	7 (29.20)	21 (41.20)	
No	17 (70.80)	30 (58.80)	
History of smoking			0.600
Yes	9 (37.50)	16 (31.40)	
No	15 (62.50)	35 (68.60)	
History of alcohol consumption			0.110
Yes	3 (12.50)	15 (29.40)	
No	21 (87.50)	36 (70.60)	
Total cholesterol (mmol/L)	4.41 ± 1.16	4.41 ± 1.22	0.999
Triglycerides (mmol/L)	1.05 (0.75, 1.65)	1.36 (1.06, 1.72)	0.113
HDL (mmol/L)	1.21 ± 0.32	1.11 ± 0.23	0.109
LDL (mmol/L)	2.57 ± 0.92	2.69 ± 1.01	0.604

BMI, body mass index; TIA, transient ischemic attack; HDL, high-density lipoprotein; LDL, low-density lipoprotein. Age and triglycerides do not follow a normal distribution and are expressed as M (Q1, Q3). TIA means transient ischemic attacks.

### Comparison of DECTA parameters for intraplaque between the STA and ATA groups

For DECTA parameters of intraplaque, in the STA group, the iodine concentration (IP_IC), effective atomic number (IP_Zeff), and CT values corresponding to the 40 keV energy spectrum curve (IP_40KH) were all greater than those in the ATA group.

The average value of the energy spectrum curve slope (IP_K) was lower in the STA group than in the ATA group, with all differences being statistically significant (*p* values <0.05) (Table 2).

**Table 2:** DECTA parameters for plaque between STA and ATA groups (*N* = 75).

DECTA parameters	STA (*n* = 24)	ATA (*n* = 51)	*P* value
IP_FF	22.90 ± 18.28	18.72 ± 19.62	0.382
IP_IC	0.39 ± 0.94	−0.18 ± 1.01	0.022*
IP_VNC	24.09 ± 22.09	24.35 ± 18.69	0.958
IP_Rho	24.55 ± 15.64	27.08 ± 17.63	0.551
IP_Zeff	7.675 (7.42, 7.84)	7.35 (7.16, 7.60)	0.003*
IP_40KH	58.63 ± 51.87	7.96 ± 39.47	<0.001*
IP_90KH	30.14 ± 15.94	28.33 ± 17.86	0.673
IP_K	−0.57 ± 0.98	0.41 ± 0.87	<0.001*

IP_, Intraplaque; FF, fat fraction; IC, iodine concentration; VNC, virtual non-contrast; Rho, electron density; Zeff, effective atomic number; 40KH, CT values at 40 keV; 90KH, CT values at 90 keV; K, the slope of the energy spectrum curve. IP_Zeff is expressed as M (Q1, Q3) due to non-normal distribution. * *p* < 0.05.

### Comparison of DECTA parameters for PVAT between the STA and ATA groups

The PVAT fat fraction (PA_FF) in the STA group was less than that in the ATA group (*p* < 0.001). In the STA group, the virtual non-contrast CT value (PA_VNC), electron density (PA_Rho), and CT values at 40 keV of the energy spectrum curve (PA_40KH) were all higher than those in the ATA group, while the slope of the energy spectrum curve (PA_K) was lower in the STA group than in the ATA group, with all differences being statistically significant (Table 3).

**Table 3:** DECTA parameters for PVAT between STA and ATA groups (*N* = 75).

DECTA parameters	STA (*n* = 24)	ATA (*n* = 51)	*P* value
PA_FF	75.41 ± 13.07	90.49 ± 16.38	<0.001*
PA_IC	−0.125 (−0.50, 0.00)	0 (0.00, 0.03)	0.075
PA_VNC	−79.25 (−92.75, −59.75)	−101 (−113.00, −90.65)	<0.001*
PA_Rho	−57.46 ± 21.52	−72.76 ± 15.33	<0.001*
PA_Zeff	6.775 (6.57, 7.01)	6.65 (6.40, 6.97)	0.233
PA_40KH	−94.93 ± 45.79	−168.39 ± 57.57	<0.001*
PA_90KH	−71.14 ± 28.03	−74.63 ± 22.98	0.570
PA_K	0.48 ± 0.83	1.88 ± 1.25	<0.001*

PA_, Perivascular Fat; FF, fat fraction; IC, iodine concentration; VNC, virtual non-contrast; Rho, electron density; Zeff, effective atomic number; 40KH, CT values at 40 keV; 90KH, CT values at 90 keV; K, the slope of the energy spectrum curve. PA_IC, PA_VNC, and PA_Zeff is expressed as M (Q1, Q3) due to non-normal distribution. * *p* < 0.05.

### LASSO regression analysis and variable selection

Nine univariate variables with *p* < 0.05 from group comparisons were included in the LASSO regression analysis. The optimal regularization parameter *λ* was determined using 10-fold cross-validation (Supplementary Figure S2). At the point of minimum cross-validation error, seven variables (IP_Zeff, IP_40KH, IP_K, PA_FF, PA_VNC, PA_Rho, PA_K) were selected for further model construction (Supplementary Figure S3). The regression coefficients for these variables were presented in Supplementary Table S1.

The logistic regression equation is as follows:

$\begin{array}{l} \mathrm{Logit}\left(P\right)=1.913+1.414\times IP\_\mathrm{Zeff}+0.023\times IP\_40KH\\ +-0.517\times IP\_K+-0.081\times PA\_FF+0.054\\ \times PA\_VNC+0.011\times PA\_\mathrm{Rho}+-1.531\times PA\_K \end{array}$

### Construction of the nomogram model for predicting SAT

**Figure 2:**
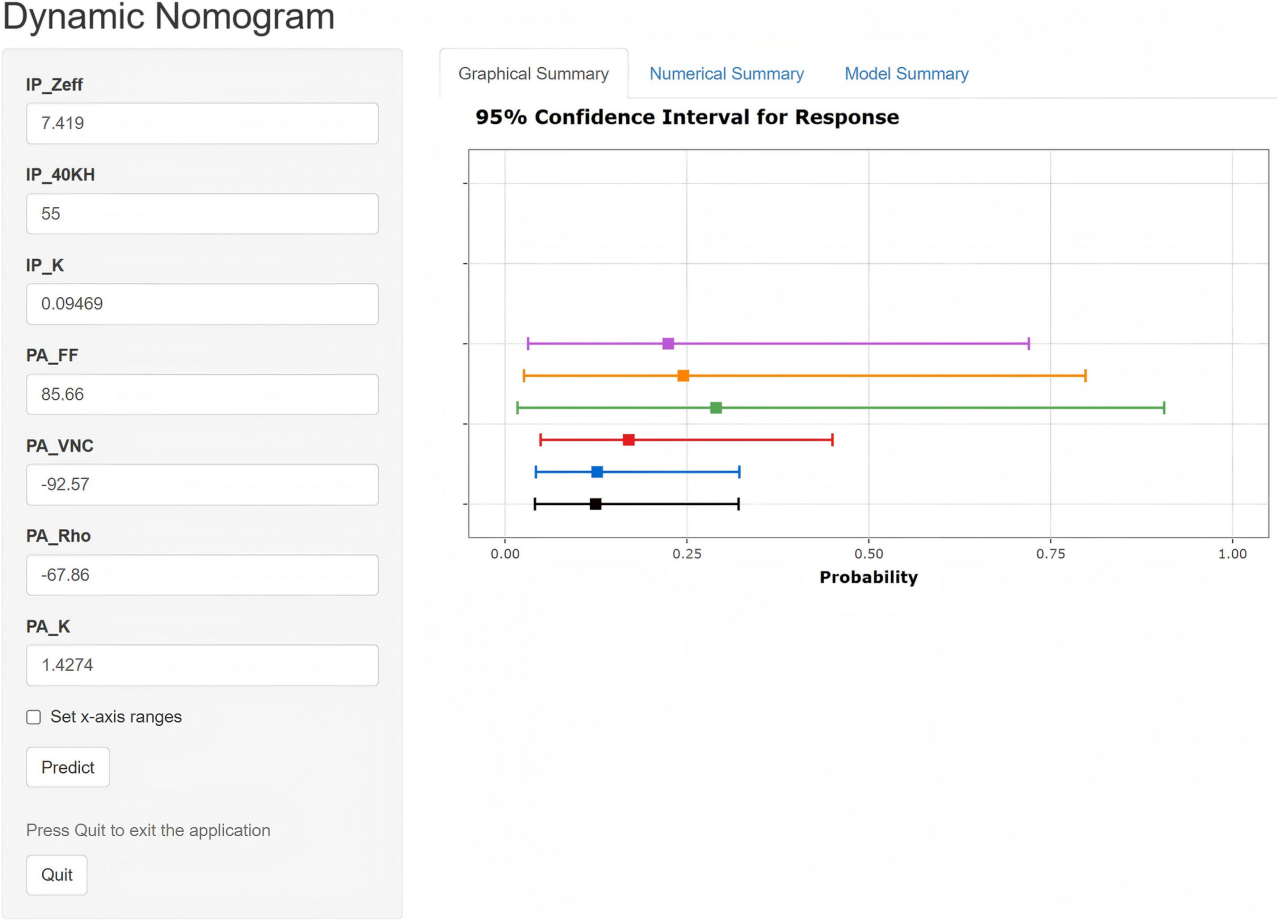
Dynamic nomogram illustrating DECTA parameters used for predicting SAT risk.

The seven variables identified through LASSO regression were incorporated into a logistic regression analysis to construct a dynamic nomogram model (Nomo_Model) for predicting the risk of acute stroke. The dynamic nomogram is accessible online at: https://dual-energy-carotid-cta.shinyapps.io/dynnomapp-1/ (Figure 2).

### Predictive performance of DECTA models

**Figure 3:**
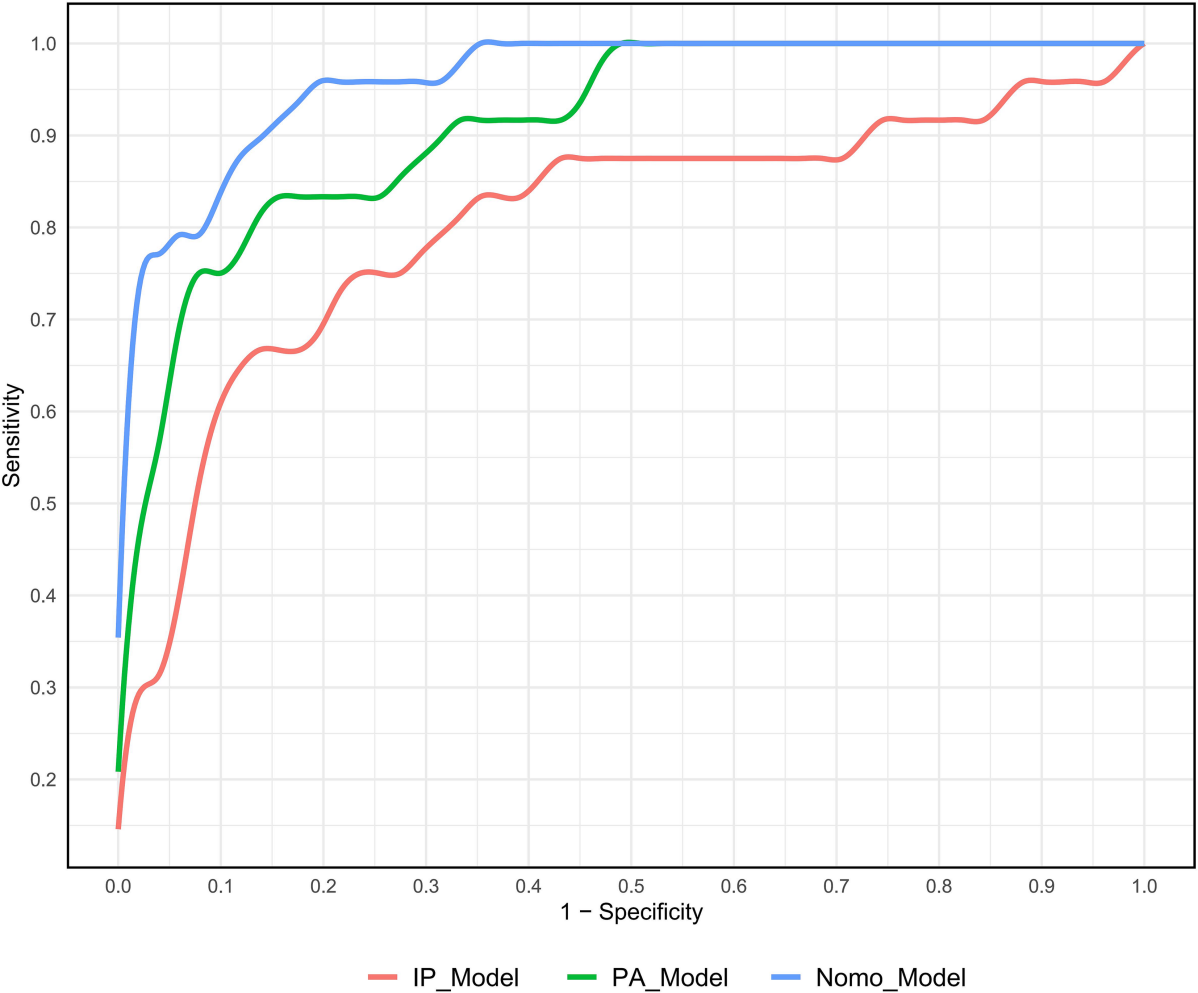
ROC curve analysis of three different DECTA models for predicting SAT.

The Nomo_Model demonstrated superior predictive performance compared to the intraplaque (IP_Model) and perivascular adipose tissue (PA_Model) models. The Nomo_Model achieved an area under the curve (AUC) of 0.962, with a sensitivity of 95.8%, specificity of 82.4%, and an accuracy of 88.0% (Table 4; Supplementary Figure S4; Figure 3).

**Table 4:** Predictive performance of DECTA parameters for SAT.

DECTA parameters	AUC (95%CI)	Sensitivity (%)	Specificity (%)
IntraPlaque (IP-)			
IP_Zeff	0.713 (0.578, 0.848)	70.8	70.6
IP_40KH	0.797 (0.674, 0.919)	66.7	88.2
IP_K	0.795 (0.676, 0.915)	75.0	82.4
**IP_Model**	0.805 (0.683, 0.926)	66.7	88.2
Perivascular fat (PA-)			
PA_FF	0.777 (0.670, 0.883)	91.7	62.7
PA_VNC	0.811 (0.711, 0.912)	75.0	72.5
PA_Rho	0.725 (0.594, 0.855)	70.8	74.5
PA_K	0.839 (0.745, 0.932)	83.3	76.5
**PA_Model**	0.913 (0.845, 0.980)	83.3	86.3
Overall combined variables			
**Nomo_Model**	0.962 (0.925, 1.000)	95.8	82.4

FF, fat fraction; IC, iodine concentration; VNC, virtual non-contrast; Rho, electron density; Zeff, effective atomic number; 40KH, CT values at 40 keV; 90KH, CT values at 90 keV; K, the slope of the energy spectrum curve; IP_Model, the plaque model; PA_Model, perivascular adipose tissue model; Nomo_Model, nomogram model.

The results of the DeLong test confirmed that the predictive efficacy of the Nomo_Model was significantly higher than that of all univariate variables, as well as the IP_Model and PA_Model (*p* < 0.05).

### Calibration and clinical decision curve analysis

The calibration curve, generated through 1,000 bootstrap iterations, demonstrated strong agreement between the Nomo_Model’s predicted probabilities and observed outcomes (Supplementary Figure S5).

**Figure 4:**
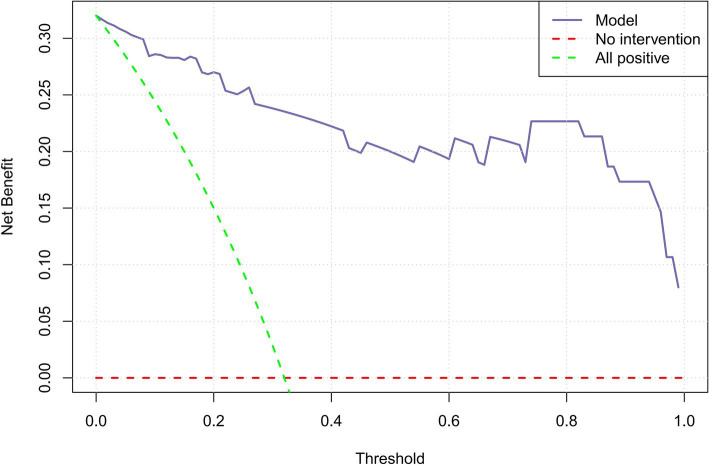
Clinical decision curve illustrating the net benefit of the Nomo_Model.

The clinical decision curve analysis indicated that the Nomo_Model provides substantial clinical benefit, particularly at intermediate and higher threshold probabilities (Figure 4).

## Discussion

In this study, we employed LASSO regression to select significant variables from dual-energy CTA-derived carotid plaque and PVAT parameters, aiming to reduce multicollinearity and prevent overfitting. These selected variables were then incorporated into a logistic regression nomogram model to predict SAT. The model demonstrated high predictive capability and clinical benefit.

Prior research has established that PVAT significantly influences atherosclerotic plaque progression. It does so by secreting inflammatory factors and active mediators that exacerbate vascular oxidation and inflammation, thereby increasing plaque vulnerability. Additionally, PVAT sequesters peroxides through an “inside-out” mechanism, which upregulates adiponectin gene expression and facilitates lipolysis. Conversely, inflammatory factors hinder the maturation of adipocyte precursors, resulting in decreased PVAT volume and heightened risk of acute cerebrovascular events (17–19). Therefore, unlike previous studies that have focused exclusively on PVAT (20), our approach integrates the dual-energy characteristics of both intraplaque and PVAT, providing a more comprehensive assessment of plaque vulnerability and enhancing predictive capabilities for acute stroke event.

In comparison to earlier investigations linking mixed-energy CTA with acute cerebrovascular events, our findings demonstrate superior predictive efficacy using dual-energy parameters. This enhancement may be attributed to the contamination of PVAT by high-density iodine contrast agents during CTA, which can compromise measurement accuracy. In contrast, virtual non-contrast images obtained from dual-energy computed tomography effectively separate iodine contrast, yielding clearer imaging (14). Moreover, low-density materials—such as loose connective tissue, fibrous tissue, lipid-rich necrotic cores, and adipose tissue—exhibit pronounced attenuation differences at low kiloelectron volts (keV), thereby improving the predictive efficacy of dual-energy CTA (21). Our results indicate that, whether in plaques or PVAT, 40 keV Hounsfield units (40KH) serve as the most reliable indicator for STA, underscoring the advantages of employing lower keV to distinguish low-density materials.

Furthermore, neovascularization within plaques is intricately linked to increased plaque activity, contributing to a heightened risk of neovascular rupture, bleeding, and inflammation (22). Several studies have documented a correlation between plaque enhancement and the extent of neovascularization (23). Specifically, in our analysis, the STA group exhibited a higher iodine concentration (IP_IC) compared to the ATA group, suggesting maybe greater neovascularization within STA plaques.

The effective atomic number (Zeff), which reflects the average atomic number of materials within a ROI, provides insights into tissue composition (11). Notably, higher density materials lead to a reduced slope of the energy spectrum curve and relatively elevated 40KH values (13). Our observations reveal that the slope of the energy spectrum curve (IP_K) in the STA group was lower than in the ATA group, while IP_40KH was higher, indicating the presence of denser components in the STA plaques. In support of this, international studies have demonstrated a robust correlation between IPH and acute cerebrovascular events (24, 25). Thus, we speculate that the STA group may harbor a higher proportion of IPH, necessitating further verification through high-resolution MRI.

Traditional CTA lacks the sensitivity required to assess tissue composition within the PVAT, limiting its ability to provide detailed information on plaque vulnerability. In contrast, DECTA enables precise measurements of iodine concentration and fat fraction in PVAT, offering critical insights into tissue characteristics associated with stroke risk. This capability facilitates a comprehensive evaluation of plaque stability and inflammation, both of which are key factors in stroke prediction.

The study underscores the value of DECTA imaging in identifying imaging biomarkers that enhance risk prediction for acute stroke. The significant differences in intraplaque and PVAT parameters between STA and ATA groups demonstrate the importance of combining both tissue-specific and vascular imaging in clinical assessments. The dynamic nomogram provides a practical and accessible tool for clinicians to estimate individual stroke risk. By incorporating multiple DECTA parameters, the model improves predictive accuracy and facilitates personalized treatment planning. Traditional clinical characteristics often fail to differentiate between STA and ATA groups. The integration of DECTA-derived parameters offers a non-invasive and precise method to evaluate plaque vulnerability and perivascular tissue characteristics, ultimately improving diagnostic outcomes.

The nomogram model provides a valuable non-invasive tool for screening patients at high risk of acute stroke. By incorporating DECTA parameters from carotid plaques and PVAT, it helps identify vulnerable plaques, allowing clinicians to prioritize high-risk patients for intensive monitoring and intervention. The model also aids in risk stratification, guiding decisions on whether additional diagnostic tests, such as MRI or cerebral angiography, are necessary. For low-risk patients, the nomogram helps optimize resource allocation and avoid unnecessary testing. Ultimately, it supports personalized treatment strategies, such as aggressive risk factor management or surgical intervention for high-risk individuals. Integrating the model into clinical decision support systems could enhance routine practice and streamline workflows in stroke centers.

While this study focused on DECTA, future research could enhance the nomogram by incorporating additional imaging techniques. High-resolution MRI could offer further details on plaque composition, such as LRNC and IPH, improving stroke risk prediction. Carotid ultrasound combined with elastography could assess plaque stiffness, providing complementary data. Including biomarkers, like lipid profiles and inflammatory markers, could also add valuable insights into the biological processes driving plaque instability. Finally, prospective validation in larger, multi-center cohorts is essential to test the model’s accuracy and generalizability across diverse clinical settings. These steps would refine the model and increase its clinical utility in patient risk assessment and management.

While DECTA offers advantages in distinguishing low-density tissues and assessing plaque vulnerability, factors such as radiation dosage, scanning parameters, and iodine contrast effects on surrounding tissues can influence its effectiveness. Excessive radiation or inconsistent parameters may introduce artifacts or variations in fat fraction and iodine concentration, compromising the accuracy of plaque and PVAT evaluations. Future studies should focus on standardizing scanning protocols to minimize these effects and ensure reproducibility. Additionally, high-resolution MRI is essential for validating DECTA findings, particularly for the accurate assessment of plaque and PVAT characteristics.

This study has several limitations: (1) the sample size is relatively small; we plan to expand this research by conducting a multi-center study with a larger cohort of patients from diverse clinical settings; (2) as a single-center retrospective clinical investigation, it lacks external validation. We intend to collaborate with other medical institutions to validate our nomogram model in independent cohorts, using standardized DECTA imaging protocols to minimize variability and enhance reproducibility; and (3) the assessment of plaques requires further validation through high-resolution MRI.

## Conclusion

DECTA imaging parameters revealed significant differences in carotid intraplaque and PVAT characteristics between the STA and ATA groups. Integrating these parameters into the nomogram (Nomo_Model) resulted in a highly accurate and clinically relevant tool for predicting acute stroke risk. However, the small sample size and lack of external validation limit the model’s generalizability.

## Data Availability

The datasets presented in this article are not readily available because they may require compliance with ethical and privacy protection requirements, and approval according to relevant agreements. Requests to access the datasets should be directed to YM, mengyankai@126.com.

## Supplementary material

SUPPLEMENTARY FIGURE S1 In the Siemens workstation's liver VNC mode **(a)** and Rho/Z mode **(b)**, ROIs were placed at the most prominent areas of the plaque (yellow circles). In the Mono E mode, the slope of the energy spectrum curve (IP_K) was acquired (white line) **(c)**. In the Siemens workstation's Liver VNC mode **(d)** and Rho/Z mode **(e)**, two ROIs were placed in the thickest PVAT gaps around the plaque (yellow circles). In the Mono E mode, the slope of the energy spectrum curve (PA_K) was calculated (white line) **(f)**.

SUPPLEMENTARY FIGURE S2 Selection of the optimal regularization parameter λ using 10-fold cross-validation. The dashed lines represent two λ values: one minimizing cross-validation error, and the other representing one standard error.

SUPPLEMENTARY FIGURE S3 Coefficient paths for variables in LASSO regression model. The dashed line represents the optimal value of λ. The asterisk (*) indicates the variables retained after selection.

SUPPLEMENTARY FIGURE S4 Confusion matrix heatmap of the Nomo_Model.

SUPPLEMENTARY FIGURE S5 Calibration curve showing the alignment between predicted and observed probabilities for the Nomo_Model.

## Glossary

LRNC: large lipid-rich necrotic core
IPH: intraplaque hemorrhage
PVAT: perivascular adipose tissue
CTA: CT angiography
DECT: Dual-energy CT
VNC: virtual non-contrast
FF: fat fraction
IC: iodine concentration
Rho: electron density
Zeff: effective atomic number
K: the slope of the energy spectrum curve
40KH: the CT value corresponding to 40 keV on the energy spectrum curve
90KH: the CT value corresponding to 90 keV on the energy spectrum curve
TR: repetition time
TE: echo time
FOV: field of view
ROI: Regions of interest
PACS: Picture Archiving and Communication System
DWI: diffusion-weighted imaging
ADC: apparent diffusion coefficient
ICA: internal carotid artery
BMI: body mass index
TIA: transient ischemic attack
ROC: receiver operating characteristic
keV: kiloelectron volts
DCA: decision curve analysis
