# Supplementary figures and images for: Development of a nomogram model for predicting acute stroke events based on dual-energy CTA analysis of carotid intraplaque and perivascular adipose tissue
Source: Front Neurol. 2025 Mar 11;16:1566395. doi: 10.3389/fneur.2025.1566395 (PMC11932918; doi:10.3389/fneur.2025.1566395)

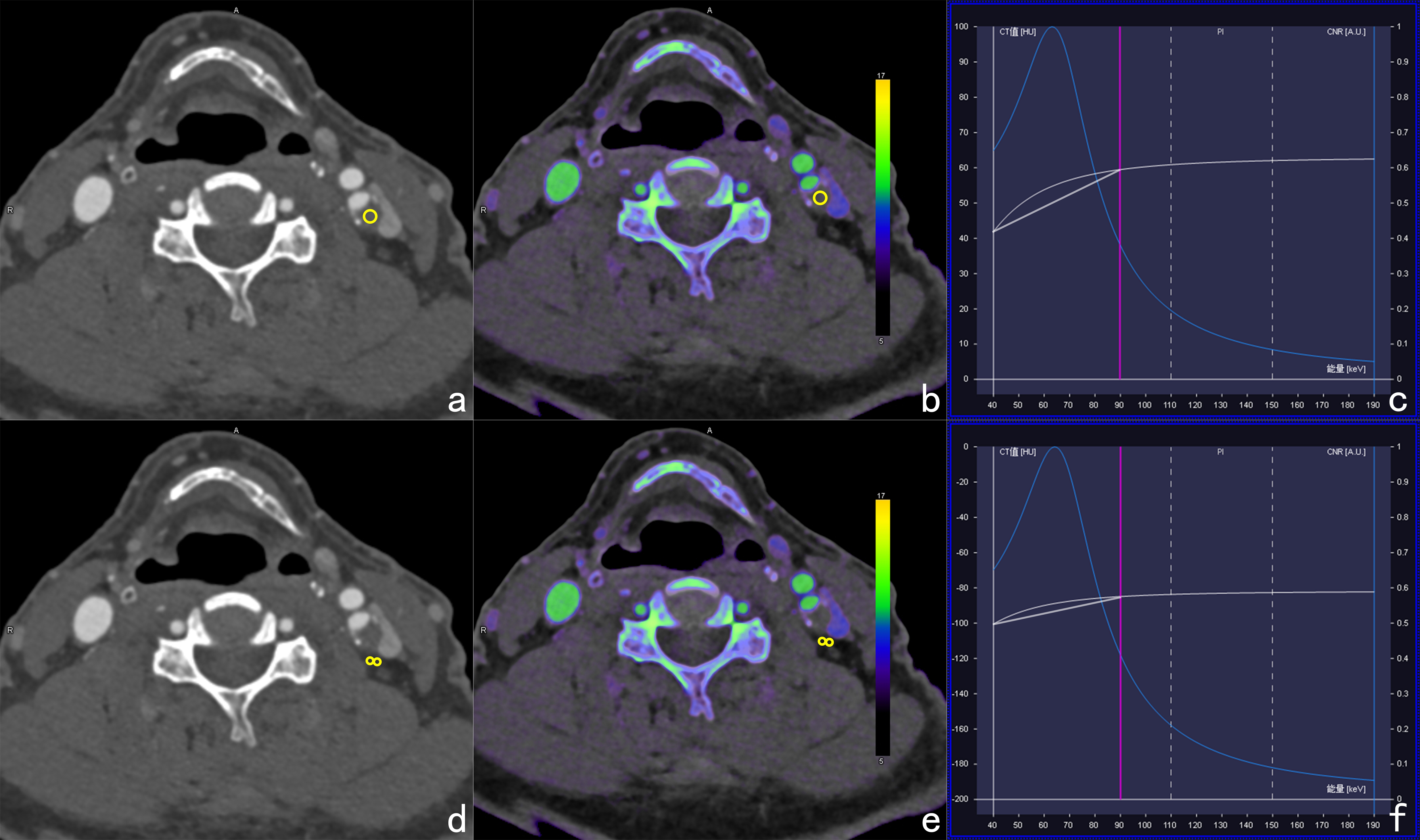

Supplement: SUPPLEMENTARY FIGURE S1 — In the Siemens workstation's liver VNC mode (a) and Rho/Z mode (b), ROIs were placed at the most prominent areas of the plaque (yellow circles). In the Mono E mode, the slope of the energy spectrum curve (IP_K) was acquired (white line) (c). In the Siemens workstation's Liver VNC mode (d) and Rho/Z mode (e), two ROIs were placed in the thickest PVAT gaps around the plaque (yellow circles). In the Mono E mode, the slope of the energy spectrum curve (PA_K) was calculated (white line) (f). [file Image_1.jpg]

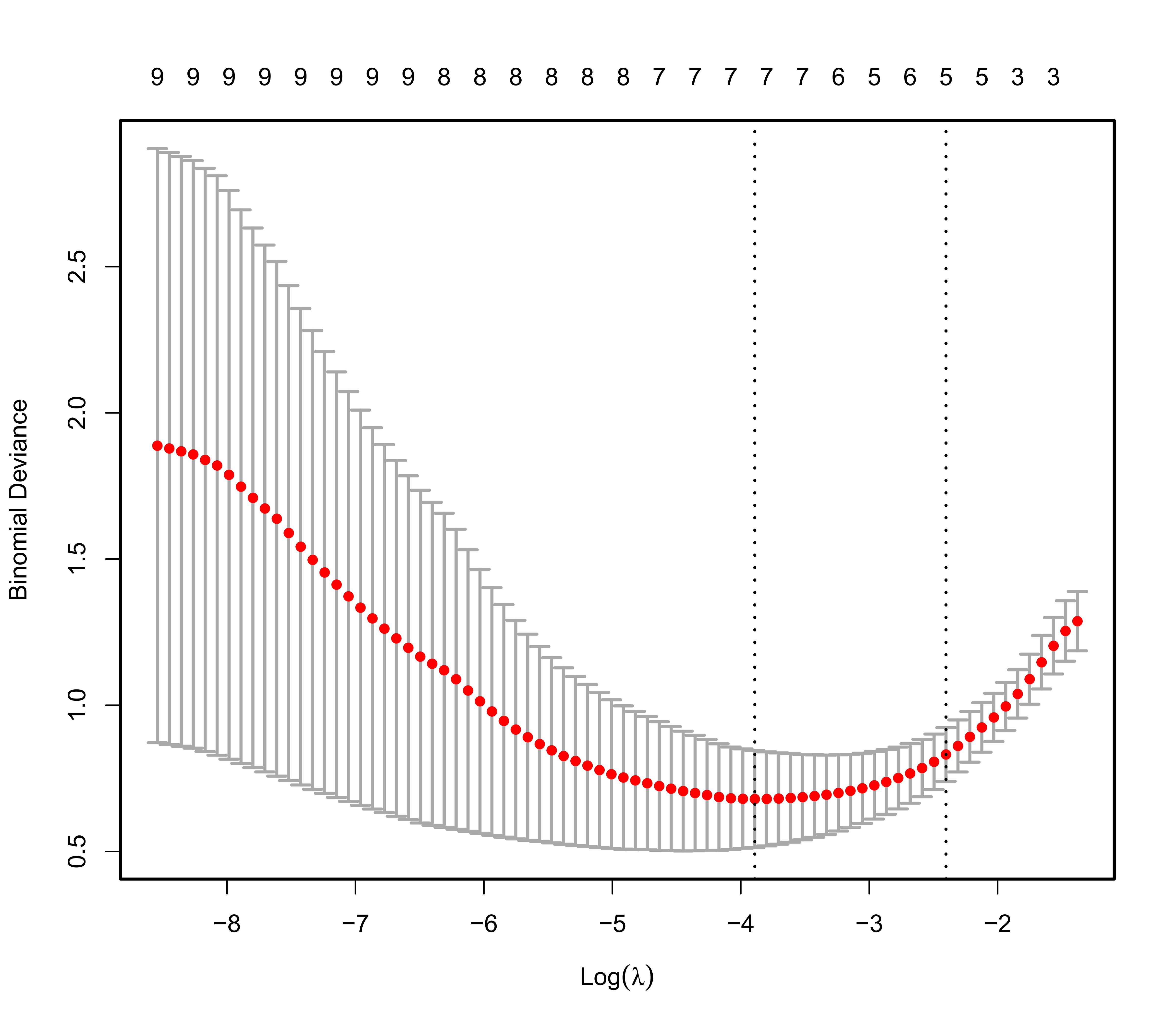

Supplement: SUPPLEMENTARY FIGURE S2 — Selection of the optimal regularization parameter λ using 10-fold cross-validation. The dashed lines represent two λ values: one minimizing cross-validation error, and the other representing one standard error. [file Image_2.jpg]

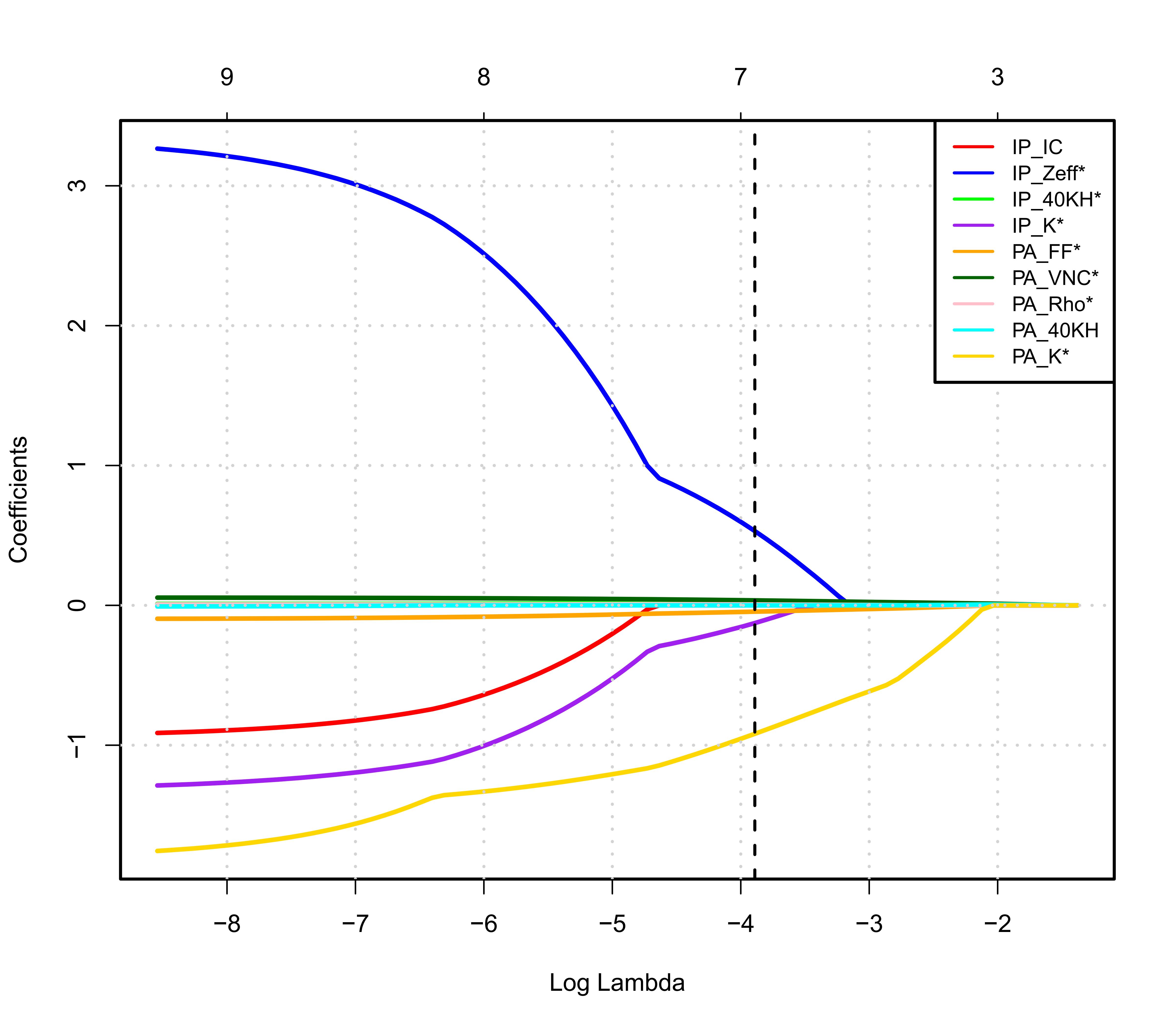

Supplement: SUPPLEMENTARY FIGURE S3 — Coefficient paths for variables in LASSO regression model. The dashed line represents the optimal value of λ. The asterisk (*) indicates the variables retained after selection. [file Image_3.jpg]

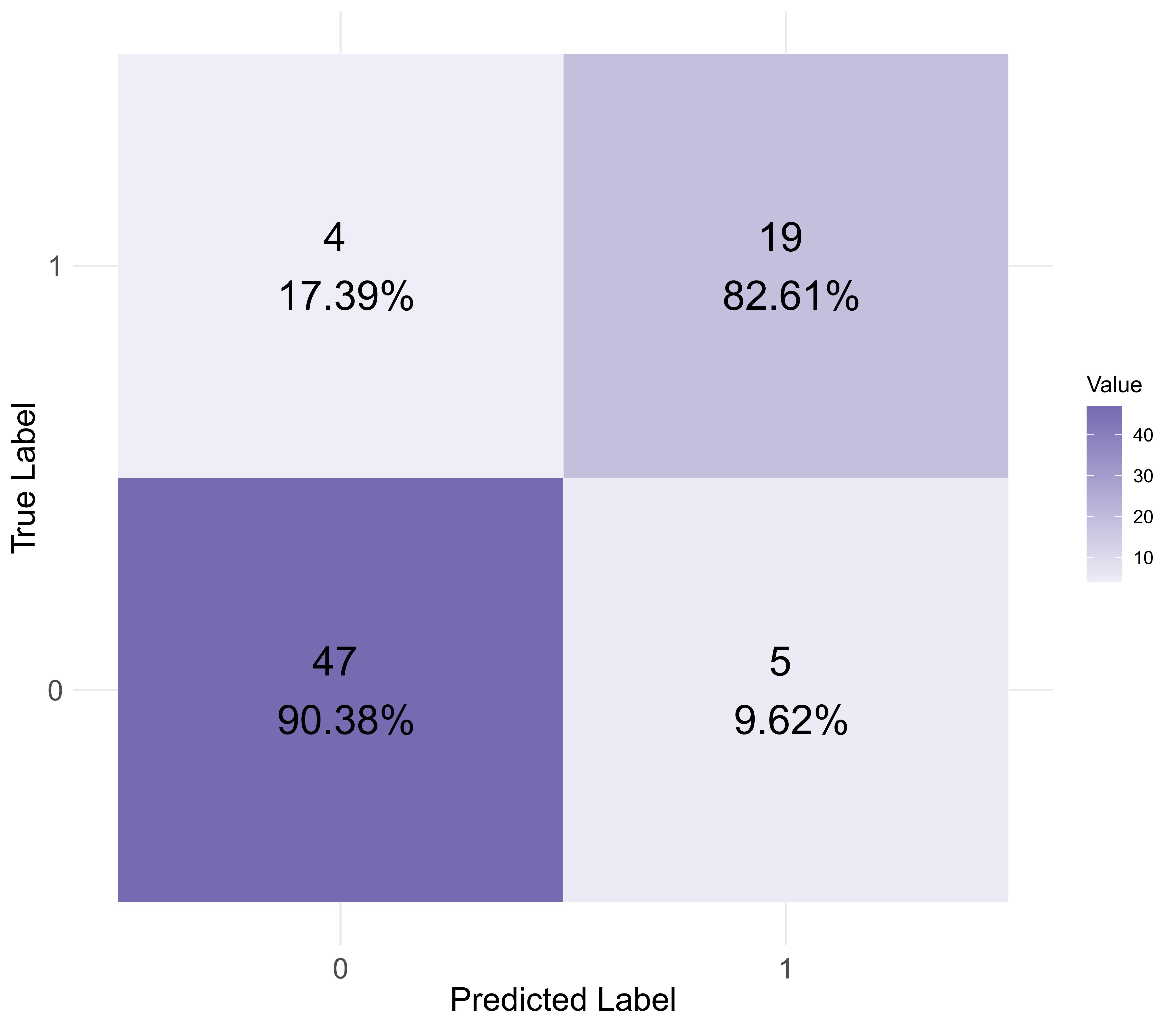

Supplement: SUPPLEMENTARY FIGURE S4 — Confusion matrix heatmap of the Nomo_Model. [file Image_4.jpg]

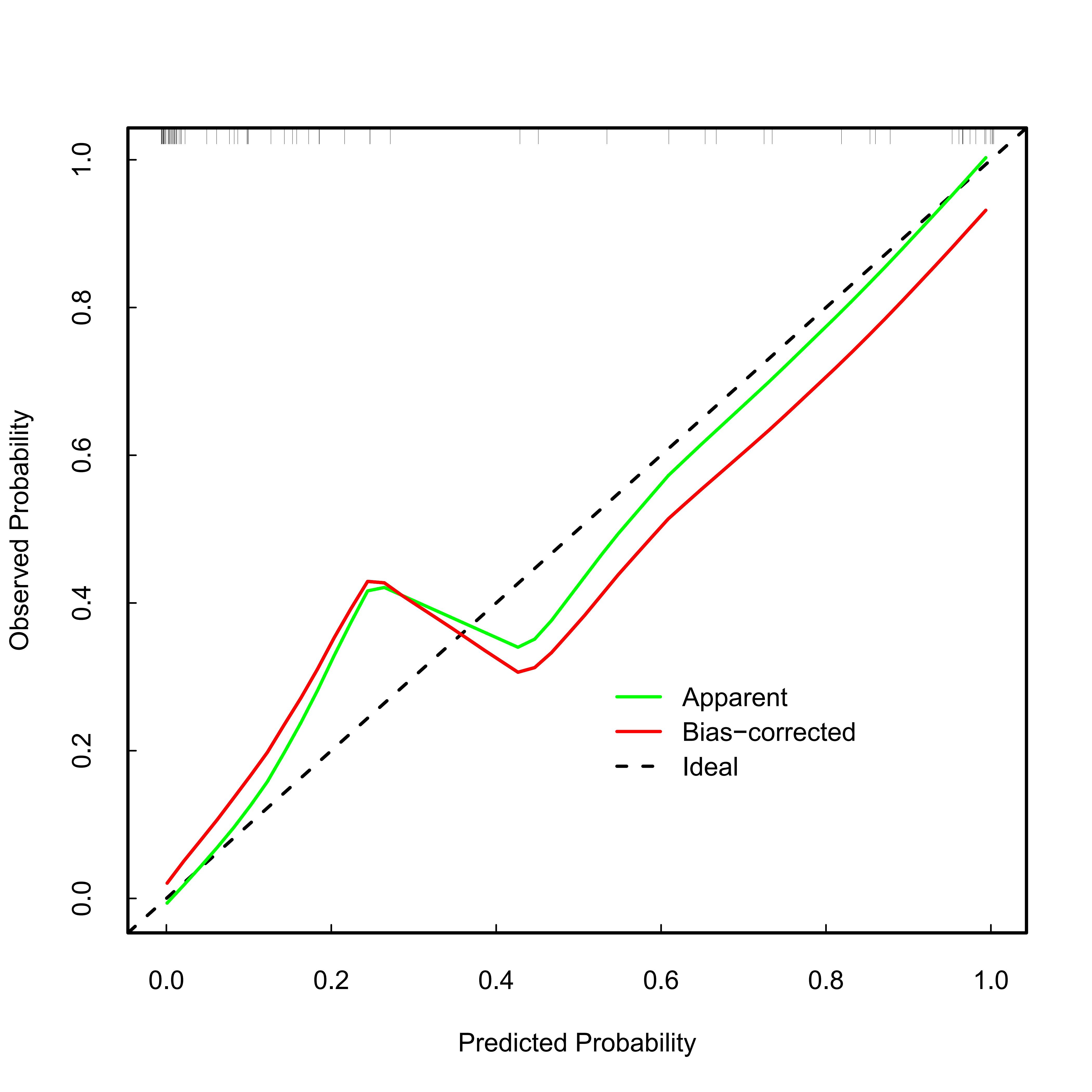

Supplement: SUPPLEMENTARY FIGURE S5 — Calibration curve showing the alignment between predicted and observed probabilities for the Nomo_Model. [file Image_5.jpg]
