# Supplementary material for: Development of a nomogram model for predicting acute stroke events based on dual-energy CTA analysis of carotid intraplaque and perivascular adipose tissue
Source: Front Neurol. 2025 Mar 11;16:1566395. doi: 10.3389/fneur.2025.1566395 (PMC11932918; doi:10.3389/fneur.2025.1566395)
Supplement: Supplementary file 6 [file Table_1.docx]

| **Supplementary Table S1** Table of LASSO-Selected Metrics | | | |
| --- | --- | --- | --- |
| Feature.Name | LASSO.Coefficient | Importance.Rank | Selected.Yes.No. |
| IP_IC | 0 |  | No |
| IP_Zeff | 0.531387327 | 6 | ***Yes*** |
| IP_40KH | 0.019778201 | 3 | ***Yes*** |
| IP_K | -0.126674832 | 4 | ***Yes*** |
| PA_FF | -0.045034091 | 5 | ***Yes*** |
| PA_VNC | 0.035582695 | 7 | ***Yes*** |
| PA_Rho | 0.006074685 | 1 | ***Yes*** |
| PA_40KH | 0 |  | No |
| PA_K | -0.918346075 | 6 | ***Yes*** |

**Note:** IP_, Intraplaque; PA_, Perivascular Fat; FF, fat fraction; IC, iodine concentration; VNC, virtual non-contrast; Rho, electron density; Zeff, effective atomic number; 40KH, CT values at 40 keV; K, the slope of the energy spectrum curve.
